# DNAJC12 Stabilizes Phenylalanine Hydroxylase and Facilitates Its Substrate‐Dependent Activation

**DOI:** 10.1096/fj.202504522RR

**Published:** 2026-07-24

**Authors:** Mary Dayne S. Tai, Trond‐André Kråkenes, Gloria Gamiz‐Arco, Christer F. Didriksen, Juha P. Kallio, Marte I. Flydal, Fernando Moro, Aurora Martinez

**Affiliations:** ^1^ Department of Biomedicine University of Bergen Bergen Norway; ^2^ Neuro‐SysMed, Department of Neurology Haukeland University Hospital Bergen Norway; ^3^ Department of Medical Genetics Haukeland University Hospital Bergen Norway; ^4^ Instituto Biofisika (EHU, CSIC) and Departamento de Bioquímica y Biología Molecular, Facultad de Ciencia y Tecnología Universidad del País Vasco EHU Leioa Spain

**Keywords:** DNAJC12, J‐domain protein, phenylalanine hydroxylase, phenylketonuria, proteostasis

## Abstract

Phenylalanine hydroxylase (PAH) is a tetrahydrobiopterin (BH_4_)‐dependent enzyme that converts L‐phenylalanine (L‐Phe) to L‐tyrosine. PAH dysfunction leads to the accumulation of L‐Phe in the blood (hyperphenylalaninemia; HPA), which may reach neurotoxic levels, resulting in phenylketonuria (PKU). PKU is associated with pathogenic variants in PAH, mostly causing misfolding and instability, leading to decreased levels of PAH protein and activity. Recently, variants in the J‐domain protein DNAJC12 have also been associated with HPA in patients, demonstrating the importance of protein homeostasis regulation for proper PAH function. DNAJC12 and PAH have previously been reported to interact, but the molecular and structural mechanisms behind complex formation have remained unclear. In this work, we show that DNAJC12 binds to PAH but presents higher affinity for its L‐Phe activated form, which resembles the conformation of unliganded tyrosine hydroxylase, a structurally and functionally related enzyme that also binds to DNAJC12. At saturation, four DNAJC12 monomers bind and stabilize the PAH tetramer, protecting it from aggregation and lowering the L‐Phe concentration necessary for substrate‐induced activation, without affecting the interaction of the enzyme with its cofactor BH_4_. Importantly, DNAJC12 also stabilizes and delays the aggregation of the PKU‐associated variant PAH‐p.R261Q. This study provides the first detailed characterization of the molecular determinants driving PAH:DNAJC12 complex formation and reveals how this interaction modulates enzyme stability and activity, and stimulates Hsc70 ATPase activity. These findings provide mechanistic insight into the pathogenic basis of DNAJC12 deficiency and identify the PAH:DNAJC12 complex as a promising therapeutic target for HPA.

## Graphical abstract

Substrate‐induced activation of PAH by L‐Phe involves dimerization of its regulatory domains (RD), with L‐Phe binding in the dimeric RD interface. The J‐domain protein DNAJC12 binds this activated conformation, stabilizing PAH and delaying aggregation, while reducing the substrate concentration required for activation. In addition, complex formation preserves PAH responsiveness to its cofactor tetrahydrobiopterin (BH4) while stimulating Hsc70 ATPase activity. The effects of DNAJC12 on PAH activity and proteostasis also provide insights into the pathogenic mechanisms of DNAJC12 disease variants.

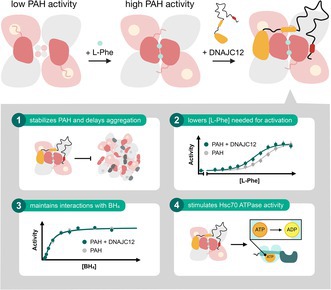

## 1 Introduction

Hyperphenylalaninemia (HPA), with phenylketonuria (PKU; MIM261600) being its most severe manifestation, is an autosomal recessive inborn error of metabolism predominantly caused by genetic variants in the *PAH* gene (NM_000277.2), which encodes the enzyme phenylalanine hydroxylase (PAH; EC 1.14.16.1). PAH converts L‐phenylalanine (L‐Phe) to L‐tyrosine (L‐Tyr) through a reaction that is dependent on non‐heme iron (Fe^2+^), molecular oxygen (O_2_) and the enzymatic cofactor (6R)‐L‐erythro‐5,6,7,8‐tetrahydrobiopterin (BH_4_) [1, 2]. Variants in *PAH* lead to deficient enzyme activity or reduced protein levels, subsequently causing an accumulation of L‐Phe in the blood and brain. Late diagnosis of patients with HPA can lead to a broad phenotypic spectrum that includes mild autistic traits, hyperactive behavior, intellectual disabilities, dystonia and parkinsonism [3].

**FIGURE 1:**
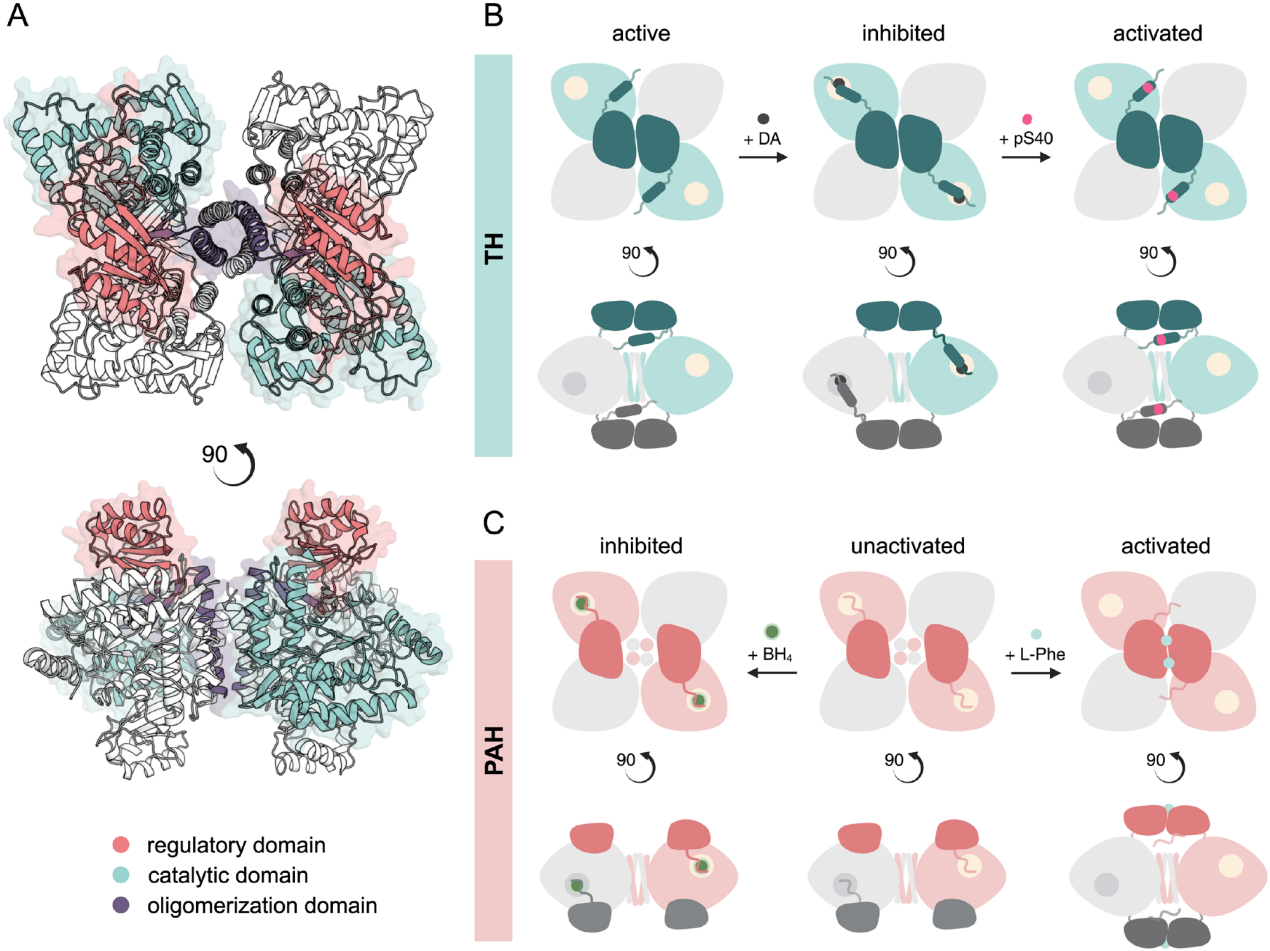
Structural features of PAH and the mechanisms for TH and PAH regulation. (A) Domain organization of PAH. X‐ray structure of PAH (PDB ID 6HYC) [5] with the regulatory domains (RD) colored in pink, catalytic domains (CD) in blue, and oligomerization domains (OD) in purple. (B) Regulation of TH by dopamine (DA) and Ser40 phosphorylation. In the active, apo, non‐phosphorylated state, the N‐terminal α‐helix (residues 39–58; dark green cylinder) is detached from the core structure. DA (black circle) binding to the active site (yellow) leads to a conformational change that fixes the N‐terminal helix, blocking DA exit and leading to feedback inhibition. Phosphorylation at Ser40 (red) leads to the detachment of the N‐terminal α‐helix and dissociation of DA, activating the enzyme [6]. (C) Regulation of PAH by BH_4_ and L‐Phe. In the unactivated apo state, the active site (yellow) is partially occluded by an autoregulatory N‐terminal region (residues 20–25; dark red), leading to low basal activity. L‐Phe (light blue circle) binding is proposed to induce RD (dark pink) dimerization from different dimers (colored subunits), with L‐Phe bound to the dimerization interface [7]. This conformational change disrupts the interactions between the RD, including the autoregulatory region, and the CD, thereby opening the active site and activating the enzyme. On the other hand, the binding of BH_4_ (green circle) to the active site stabilizes the interaction between the autoregulatory region and the CD, and inhibits the enzyme [5].

PAH is a homotetrameric hepatic enzyme belonging to the aromatic amino acid hydroxylase family (AAAHs), a group of iron‐ and BH_4_‐dependent enzymes that hydroxylate their respective amino acid substrates [4]. This family comprises PAH, tyrosine hydroxylase (TH), and tryptophan hydroxylases 1 and 2 (TPH1 and TPH2). Each 51‐kDa PAH subunit contains an N‐terminal regulatory domain (RD; residues 1–110), a catalytic domain (CD; residues 111–410) responsible for hydroxylating L‐Phe, and a C‐terminal oligomerization domain (OD; residues 411–452) [5] (Figure 1A). Unlike TH, whose activity is regulated primarily by its end product dopamine (Figure 1B), PAH displays positive cooperativity in response to increasing concentrations of its substrate L‐Phe [8, 9] (Figure 1C). The conformational transition induced by L‐Phe is slow [10] and leads to the dimerization of the RDs on opposite sides of the CD and ODs [11, 12], with L‐Phe binding at the dimerization interface [7].

Currently, more than 3000 PAH variants are registered in the BIOPKU database (http://www.biopku.org/). Most PKU‐causing variants are missense (33.7%), deletions and splicing alterations and, recently, most missense variants have been classified using structural analysis and phenotypic data [13]. The major pathogenic mechanism associated with PKU variants is their instability, leading to decreased activity and increased degradation [14, 15, 16], and PKU is traditionally viewed as a loss‐of‐function disease. Nevertheless, the characterization of a mouse model harboring one of the most common PKU‐associated variants, PAH‐p.R261Q, reveals a gain‐of‐function pathogenic mechanism, where the unstable and misfolded PAH protein forms toxic amyloid‐like aggregates [17].

Although most patients with HPA have variants in *PAH*, approximately 2% of cases are related to deficiencies in the synthesis or regeneration of the cofactor BH_4_ [18]. An even smaller proportion of HPA cases have more recently been attributed to biallelic mutations in the gene encoding the cochaperone DNAJC12 in patients without any variants in *PAH* or other genes related to BH_4_ synthesis or regeneration [19, 20, 21]. DNAJC12 is a specific interaction partner of the AAAHs [22, 23, 24], including PAH, and thus DNAJC12 deficiency leads to impaired AAAH proteostasis and reduced cellular levels of these enzymes [19, 25]. DNAJC12 is a class C J‐domain containing protein (JDP), that recognizes and binds to specific clients and presents them to Hsp70 for the regulation of client protein homeostasis [26]. Furthermore, an Hsp70‐independent holdase activity has recently been suggested for DNAJC12 towards its client TH, where the cochaperone increased the stability of TH and prevented its aggregation over time in vitro [23].

DNAJC12 is a monomeric protein of 198 amino acids comprising a J‐domain (JD; residues 14–76) that is essential for its interaction with Hsp70, a long linker region that includes a linker‐helix (residues 87–100), and a highly conserved C‐terminal domain (CTD; residues 176–198), which, as demonstrated for TH, is involved in client binding [23]. DNAJC12 recognizes and binds to the dimerized RDs of TH [23] and is expected to recognize the same domain in the other AAAHs, despite the RD being the least conserved domain within this family of enzymes. However, the exact molecular mechanisms by which PAH and DNAJC12 interact, and the biological consequences of this interaction, are unknown.

Here, we report that DNAJC12 binds to PAH, preferentially to its allosterically L‐Phe activated state, characterized by RD dimerization. Despite sequence divergence in the RDs of AAAHs, DNAJC12 recognizes PAH by the CTD. This interaction stabilizes PAH, particularly the RD, and lowers the L‐Phe concentration needed for enzyme activation without affecting the positive cooperativity for L‐Phe or its interactions with BH_4_. DNAJC12 also binds the L‐Phe activated state of the variant PAH‐p.R261Q, increases its activity at lower L‐Phe concentrations, and delays aggregate formation in vitro. These results underscore the regulatory role of DNAJC12 on PAH activity and stability, highlighting the PAH:DNAJC12 interaction as a potential therapeutic target for HPA and PKU.

## 2 Materials and Methods

### 2.1 Plasmids

The pETMBP1a/*DNAJC12* plasmid encoding human wild‐type (WT) DNAJC12, as well as the derivative plasmids encoding truncated DNAJC12 variants DNAJC12(1–190) and DNAJC12(1–174 or Trp175Ter) [23], as well as the pMAL‐c5x/*PAH* plasmid encoding human PAH‐WT were previously available in the laboratory. Plasmids encoding PAH variants containing the RD (PAH(33–112)) or CD + OD (PAH(103–452)), and the disease‐associated PAH variant PAH‐p.R261Q were derived from the pMAL‐c5x/*PAH* plasmid (GenScript). The cDNAs of Apg2 (HSPH2), Hsc70 (HSPA8), DNAJA2 and DNAJB1 (Addgene) were cloned into the pE‐SUMO vector (LifeSensors).

### 2.2 Protein Purification

DNAJC12‐WT and variants were expressed in *E. coli* BL21‐CodonPlus(DE3)‐RIL and purified as described by Tai et al. [23]. PAH‐WT and variants were purified from *E. coli* K12 TB1 cells (New England BioLabs) using a protocol previously described by Flydal et al. [5]. Recombinant proteins containing N‐terminal His and SUMO tags (Apg2, Hsc70, DNAJA2, DNAJB1) were expressed in *E. coli* BL21 CodonPlus(DE3)‐RIL or Rosetta(DE3) as previously described by Cabrera et al. [27] and Velasco‐Carneros et al. [28]. The C‐terminal peptide DNAJC12(176–198) was purchased from GenScript.

### 2.3 Analytical Size Exclusion Chromatography (SEC)

Samples containing 20 μM (subunit) PAH without or with the addition of 20 μM DNAJC12 were prepared and analyzed on a Superdex 200 Increase (1.0 × 30 cm) column at a flow rate of 0.5 mL/min at 4°C, with a 20 mM Na‐Hepes pH 7.0, 200 mM NaCl or with the addition of 1 mM L‐Phe.

### 2.4 Purification of the PAH:DNAJC12 Complex

The PAH:DNAJC12 complex was purified using SEC on a Superdex 200 Increase (1.0 × 30 cm) column by combining PAH and DNAJC12 proteins in a 4:8 PAH:DNAJC12 (subunits) molar ratio and collecting the fractions corresponding to the peak shifted to an earlier elution volume. The run was done at a flow rate of 0.5 mL/min at 4°C, with a 20 mM Na‐Hepes pH 7.0, 200 mM NaCl, 1 mM L‐Phe.

### 2.5 Native PAGE

Samples with 0.2 mg/mL DNAJC12, PAH or purified PAH:DNAJC12 complex in 20 mM Na‐Hepes pH 7.0, 200 mM NaCl were diluted 1:1 with native PAGE Sample Buffer (Bio‐Rad) and loaded into a 10% Mini‐PROTEAN TGX Precast Protein Gel (Bio‐Rad). The gel was run at 140 V for 3 h at 4°C with running buffer (25 mM Tris pH 8.3, 192 mM glycine), prior to visualization using 0.08 mM Coomassie brilliant blue G‐250 (Bio‐Rad) with 5 mM HCl. For the native PAGE experiments monitoring PAH:DNAJC12 or TH:DNAJC12 complex formation at varying DNAJC12 concentrations, samples were prepared the same way, where 0.2 mg/mL PAH or TH was combined with DNAJC12 at increasing molar ratios (4:1, 4:2 or 4:4).

### 2.6 Immunoblotting

Proteins were transferred from the PAGE gel onto a polyvinylidene difluoride membrane (Bio‐Rad) using the Transblot Turbo Transfer System (Bio‐Rad) at 25 V for 3 min. The membrane was blocked with blocking solution (5% w/v skimmed milk powder, 1× Tris‐buffered saline (TBS), 1% Tween 20). Primary antibody incubation was carried out overnight using a rabbit anti‐DNAJC12 antibody (1:10000; ABCAM, cat. no. AB167425) or anti‐PAH antibody (1:10000; ABCAM, cat. no. AB178430). The membrane was subsequently washed to reduce unspecific binding using washing buffer (1× TBS, 0.1% Tween 20) before incubation with goat anti‐rabbit antibody (1:1000 and 1:5000 for DNAJC12 and PAH detection, respectively; Bio‐Rad, cat. no. 1706515) for 1 h. The membrane was washed again prior to visualization using enhanced Luminata Immobilon Crescendo Western horse radish peroxidase substrate (Merck Millipore) and the ChemiDoc XRS+ System (Bio‐Rad).

### 2.7 Bio‐Layer Interferometry

Bio‐layer interferometry (BLI) was conducted using an OctetRED96 platform (FortéBio) with streptavidin (SA) biosensors (Sartorius). For PAH or TH immobilization on the biosensor, the proteins were biotinylated using EZ‐Link NHS‐PEG4‐Biotin (Sigma Aldrich) according to the manufacturer's instructions and diluted to 50 μg/mL in assay buffer (1× phosphate‐buffered saline (PBS), 0.02% Tween‐20, 0.5 mg/mL BSA) with or without 1 mM L‐Phe. DNAJC12 was also prepared in the assay buffer at varying concentrations (0.01, 0.1, 0.5, 1, 2, and 4 μM) in the presence or absence of 1 mM L‐Phe. The samples were then dispensed into a 96‐well flat‐bottom polypropylene plate (Greiner), maintained at 25°C, and agitated at 1000 rpm during the assay. Prior to each experiment, the biosensors were soaked in 1× PBS for 1–3 h, followed by equilibration in assay buffer for 30 s, PAH loading for 300 s, and re‐equilibration in assay buffer for 120 s. The binding of DNAJC12 to immobilized PAH was monitored for 600 s, followed by a final equilibration in assay buffer for 300 s. Data were processed using the manufacturer's software (Octet Data Analysis HT). Signals from the zero‐concentration sample were subtracted from those obtained for each functionalized biosensor, plotted against the respective analyte concentrations, and fitted to a one‐site binding (hyperbola) non‐linear regression model to derive the K_D_ values.

### 2.8 SEC Coupled With Multi‐Angle Light Scattering

The sizes of PAH (2 mg/mL), pre‐purified PAH:DNAJC12 complex (2 mg/mL), MBP‐PAH(RD) (4 mg/mL) and MBP‐PAH(RD):DNAJC12 complexes (4 mg/mL MBP‐PAH(RD); 4 mg/mL DNAJC12) were determined by SEC coupled with multi‐angle light scattering (SEC‐MALS). Samples containing the MBP‐PAH(RD) were prepared in 20 mM Na‐Hepes pH 7.0, 200 mM NaCl, supplemented with 1 mM L‐Phe, while samples containing full‐length PAH were prepared in the same sample buffer without 1 mM L‐Phe supplementation. All samples were filtered using Corning Costar Spin‐X (0.22 μm) plastic centrifuge tube filters (Merck) prior to sample application. The proteins were analyzed using a Superdex 200 Increase (1.0 × 30 cm) column connected to an iSeries LC‐2050 (Shimadzu) HPLC system coupled to a RefractoMax 520 module (ERC GmbH) to measure the refractive index and determine concentration, and a mini‐DAWN TREOS detector (Wyatt Technology) to measure light scattering. Data processing and molar mass estimation were performed using the Astra software (Wyatt).

### 2.9 Differential Scanning Fluorimetry

Thermal denaturation curves of PAH alone or in the presence of DNAJC12, were obtained at different L‐Phe concentrations using differential scanning fluorimetry (DSF). Samples with 1.92 μM (subunit) PAH alone or in presence of 3.84 μM DNAJC12 (4:8 PAH:DNAJC12 subunit ratio) were prepared with 2× ammonium iron (II) sulphate (FAS), 5× SYPRO Orange dye (Merck) in the presence or absence of L‐Phe at varying concentrations (0–10 mM) prior to loading into a 384‐well plate (Corning). The plate was heated step‐wise from 25°C to 90°C at a heating rate of 2°C/min using a LightCycler 480 Real‐Time PCR System (Roche Applied Science). The thermal denaturation of PAH was monitored by detecting the increase in SYPRO Orange fluorescence (*λ*_ex_ = 465 nm, *λ*_em_ = 610 nm) upon its binding to exposed hydrophobic patches. Data analysis was done using HTSDSF Explorer [29] to obtain the melting temperatures of the regulatory (*T*_m1_) and catalytic (*T*_m2_) domains. Additional binding experiments with the DNAJC12(176–198) C‐terminal peptide were conducted similarly, monitoring PAH (1.92 μM) unfolding in the presence of increasing peptide concentrations (0–16 μM).

### 2.10 Dynamic Light Scattering

PAH aggregation was monitored in vitro by dynamic light scattering (DLS) using the Zetasizer Nano ZS instrument (Malvern Panalytical). Samples were prepared to contain 10 μM PAH (subunit) alone or with 10 μM DNAJC12 in 20 mM Na‐Hepes pH 7.0, 200 mM NaCl, or with an addition of 1 mM L‐Phe. The samples were centrifuged at 16000 x g for 5 min and filtered using Corning Costar Spin‐X (0.22 μm) plastic centrifuge tube filters (Merck) prior to sample loading into UV cuvettes (Merck). Protein aggregation was analyzed by monitoring the change in the average particle size or Z‐average value (diameters in nanometers; d.nm) of the protein samples over a 115‐min period at 37°C. Light scattering was measured every 2 min using a He‐Ne laser at 683 nm with a fixed scattering angle of 173°. PAH‐WT samples containing L‐Phe were incubated for an additional 30 min at 37°C prior to collecting measurements.

### 2.11 ATPase Measurements

Steady‐state ATPase assays were performed at 30°C in 40 mM K‐Hepes pH 7.6, 50 mM KCl, 5 mM Mg acetate, 2 mM DTT and an ATP regeneration‐system (0.3 mM NADH, 3 mM phosphoenolpyruvate, 12.5 ng/mL pyruvate kinase, 0.017 mg/mL lactate dehydrogenase) in a final volume of 200 μL. Protein concentrations were 2 μM Hsc70, 0.4 μM Apg2, 1 μM JDPs, 1 μM PAH or PAH‐p.R261Q. PAH and PAH‐p.R261Q were initially incubated for 15 min at room temperature with 1 mM L‐Phe, where indicated, followed by a further incubation for 15 min after addition of DNAJC12 or DNAJA2, or DNAJB1. The reaction was started after addition of Hsc70, Apg2 and 2 mM ATP (final concentration). ATP consumption was measured following the NADH decay by continuous measurement of the absorbance at 340 nm for 1 h using a Synergy HTX plate reader (BioTek) and transparent 96‐well flat bottom plates (Sarstedt). The ATPase rates (μmol ATP/min) were calculated from the slopes of the A340 decay curves over the selected time intervals that showed a linear absorbance decline, using the extinction coefficient of NADH (ε340 = 6220 M^−1^ cm^−1^).

### 2.12 PAH Activity Assay

PAH activity was assayed at 25°C by continuously measuring L‐Tyr production based on the increase in fluorescence intensity (excitation wavelength 274 nm and emission wavelength 304 nm) using a Spark 20 M microplate reader (Tecan). The standard reaction buffer containing 0.04 mg/mL catalase, 10 μM FAS, 1 mM L‐Phe and 100 mM Na‐Hepes pH 7.0 was added to sample wells on a black flat‐bottom 96‐well microplate (Greiner). PAH was added to a final concentration of 0.005 mg/mL with 0.05% BSA in the assay and preincubated for 3 min at 25°C to be activated by L‐Phe, without or with 0.01 mg/mL DNAJC12. The reaction was started by adding 75 μM BH_4_ with 2 mM DTT and L‐Tyr production was measured in real‐time for 15 min. To determine activity as a function of substrate, the concentration of L‐Phe in the reaction mix was varied from 0 to 1000 μM and BH_4_ was kept constant at 75 μM. Similarly, to determine activity as a function of the cofactor, the BH_4_ concentration was varied from 0 to 150 μM and L‐Phe was kept constant at 1 mM. All given concentrations refer to the final concentration in a 50 μL reaction mixture. Fluorescence intensity was recorded for all enzyme activity measurements, and the reaction velocity was converted to enzyme activity (nmol Tyr/min/mg protein) using L‐Tyr standards.

### 2.13 Statistics and Reproducibility

DSF dose response and PAH activity assay results were fitted to a nonlinear regression curve using the four‐parameter dose–response model,

$$v=V_{\min}+\frac{\left(V_{\max}-V_{\min}\right){\left[S\right]}^{h}}{{\mathrm{EC}}_{50}^{h}+{\left[S\right]}^{h}}$$

For the activity assays, *v* is the observed enzyme velocity, *V*_min_ and *V*_max_ are plateaus in the velocity, [*S*] is the substrate concentration and EC_50_ is the substrate concentration that gives half maximal velocity, and *h* is the Hill coefficient (GraphPad Prism 10.4.0). Values are given as the mean ± SD of three independent experiments done with technical triplicates. Accordingly, *v* is the observed enzyme *T*_m_, *V*_min_, and *V*_max_ are plateaus in the *T*_m_, while the EC_50_ represents the substrate concentration that gives half maximal stabilization, and are given as the mean ± SD of three independent experiments for the DSF dose response assays. For the ATPase activity assays, at least three samples were prepared and measured prior to performing multiple comparisons using one‐way analysis of variance (ANOVA) and a post hoc Tukey HSD test. Results were considered significantly different when *p* < 0.05.

## 3 Results

### 3.1 Four DNAJC12 Monomers Bind to the Native PAH Tetramer in the Presence of L‐Phe

**FIGURE 2:**
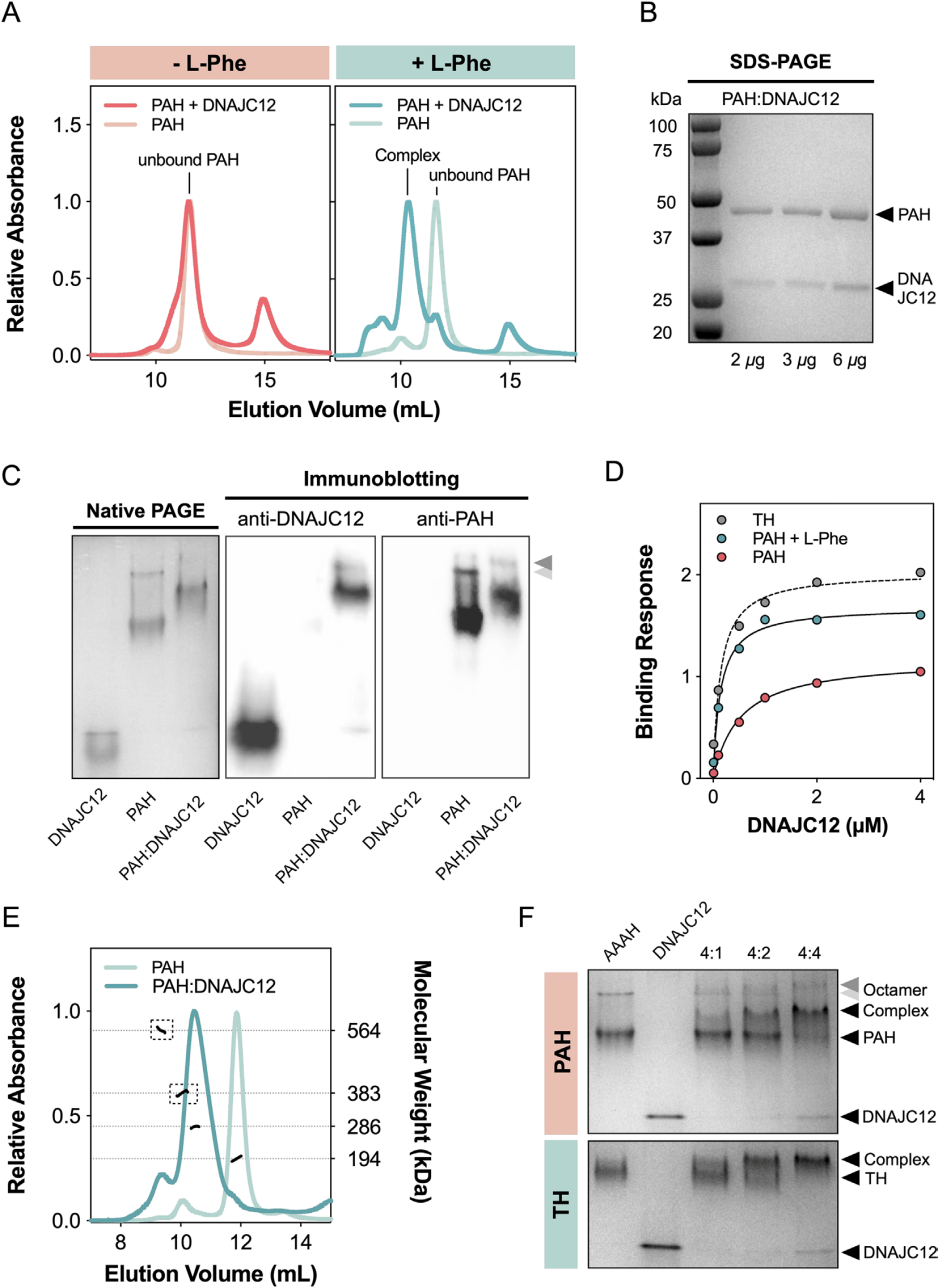
DNAJC12 binds to the activated form of PAH at 4(PAH):4(DNAJC12) stoichiometry. (A) SEC chromatograms of PAH alone and with DNAJC12, with and without L‐Phe. The presence of DNAJC12 (20 μM) shifts the elution of a small proportion of tetrameric PAH (20 μM subunit), indicating complex formation, but PAH mostly remains unbound. In the presence of 1 mM L‐Phe, DNAJC12 (20 μM) up‐shifts the elution of tetrameric PAH (20 μM subunit), indicating efficient complex formation. (B) SDS‐PAGE analysis of the purified PAH:DNAJC12 complex. SDS‐PAGE of the complex (2, 3, or 6 μg protein), showing the co‐elution of DNAJC12 (∼27 kDa) and PAH (∼48 kDa). (C) Native PAGE (left) and immunoblot (middle and right) analyses of the PAH:DNAJC12 complex and controls. PAH alone results in the detection of two species: a predominant tetrameric species and a slower‐migrating higher‐order species (light gray arrow), likely corresponding to tetrameric and octameric PAH, respectively (see also Table 1, in this figure E,F). The purified PAH:DNAJC12 complex migrates slower than PAH alone through the native gel (left) and also displays two distinct bands likely corresponding to tetrameric and octameric (dark gray arrow) PAH:DNAJC12 complexes. Using immunodetection with antibodies against DNAJC12 (middle) and PAH (right), both proteins are detected in these two bands, confirming complex formation. (D) BLI analyses of full‐length DNAJC12‐concentration dependent binding to PAH, with or without L‐Phe, as compared to TH. L‐Phe increases the affinity of DNAJC12 for PAH by more than 3‐fold (K_D_ = 139 ± 19 nM with L‐Phe; blue symbols; and K_D_ = 508 ± 59 nM without L‐Phe; red symbols), to similar levels as that observed for the TH:DNAJC12 interaction (K_D_ = 138 ± 34 nM). (E) Determination of PAH:DNAJC12 stoichiometry by SEC‐MALS. Analysis of the PAH:DNAJC12 complex (200 μg; dark blue) and PAH (200 μg; light blue) by SEC‐MALS provided an estimated size of 286.2 ± 1.8 kDa for the complex, consistent with the binding of four DNAJC12 monomers to each PAH tetramer (194.1 ± 4.6 kDa). In addition, octameric PAH (383.1 ± 5.4 kDa) is also detected and forms a complex with 8 DNAJC12 monomers (564.0 ± 4.6 kDa) (stippled boxes). See Table 1 for additional values. (F) Native PAGE analyses of PAH and TH with varying amounts of DNAJC12. Addition of varying amounts of DNAJC12 (4:1, 4:2, and 4:4 AAAH subunit:DNAJC12 ratio) to a constant amount of PAH or TH shows that a proportion of PAH remains unbound even at a 4:4 ratio, while all TH has bound under the same conditions. A larger PAH oligomer (light gray arrow) is also detected and increases in size, consistent with the formation of the octameric PAH:DNAJC12 complex (dark gray arrow).

The TH:DNAJC12 complex that includes two DNAJC12 molecules per TH tetramer was previously obtained by combining TH and DNAJC12 in a 4:4 subunit ratio, followed by analysis and purification using size exclusion chromatography (SEC) [23]. Unlike TH, which fully forms the complex under these conditions, PAH does not, and only a small proportion of PAH shows a shifted elution profile, indicating the presence of some PAH:DNAJC12 complex (Figure 2A; left).

While PAH and TH share sequence and structural conservation to a large degree, the unliganded states of both proteins display different structural features in the resting state. In particular, the PAH‐RDs are monomeric [5, 11], while the TH RDs form dimers on the opposite sides of the CD + OD [6] (Figure 1B,C). However, in the presence of its substrate L‐Phe, the PAH‐RDs dimerize [7, 12]. As DNAJC12 was recently described to bind around the dimeric RDs of TH [23], we hypothesized that the large conformational change associated with enzyme activation by L‐Phe, leading to dimerization of the RDs, could improve the binding of DNAJC12 to PAH. Thus, we repeated the binding experiment supplementing the running and sample buffers with 1 mM L‐Phe, a concentration that activates human PAH [8]. Although L‐Phe induces dimerization of the RDs, this local rearrangement does not alter the overall tetrameric assembly of full‐length PAH, resulting in an unchanged SEC elution profile with or without L‐Phe (Figure 2A). In the presence of both L‐Phe and DNAJC12, however, a large up‐shift in the elution profile of PAH was observed (Figure 2A; right), indicating the efficient formation of the PAH:DNAJC12 complex. The presence of the complex in this fraction was demonstrated by PAGE in denaturing and non‐denaturing conditions. SDS‐PAGE analysis revealed two bands corresponding to DNAJC12 (23.5 kDa) and PAH (52 kDa), confirming the co‐elution of the two proteins (Figure 2B), and analysis of the same sample using native PAGE showed slower migration through the polyacrylamide matrix, as compared to PAH alone (Figure 2C; left). Subsequent immunoblotting of the native PAGE gel with anti‐DNAJC12 antibodies resulted in the detection of DNAJC12 in the DNAJC12 control and in the band corresponding to the PAH:DNAJC12 complex, but not in the PAH control (Figure 2C; middle). Conversely, anti‐PAH antibodies detected PAH in the PAH control and in the complex sample, but not in the DNAJC12 control (Figure 2C; right). Together, these results confirm co‐migration of PAH and DNAJC12 and support stable complex formation.

The effect of L‐Phe on the affinity between PAH and DNAJC12 was then investigated by bio‐layer interferometry (BLI). The binding response between immobilized biotinylated PAH on streptavidin biosensors and varying DNAJC12 concentrations was measured with and without 1 mM L‐Phe. In agreement with the SEC results (Figure 2A), we found that L‐Phe increases the affinity of DNAJC12 for PAH by more than 3‐fold (from K_D_ = 508 ± 59 nM without L‐Phe to K_D_ = 139 ± 19 nM with L‐Phe), to a similar level as the affinity between DNAJC12 and unliganded TH (K_D_ = 138 ± 34 nM; Figure 2D). Furthermore, the stoichiometry of the PAH:DNAJC12 complex was determined by SEC coupled with multi‐angle light scattering (SEC‐MALS). While PAH alone presented the expected mass for the tetrameric enzyme (194.1 ± 4.6 kDa), the complex was estimated to be 286.2 ± 1.8 kDa, ~92 kDa larger than PAH alone (Figure 2E). As each DNAJC12 monomer is 23.5 kDa [23], these results indicate that four DNAJC12 monomers bind to each PAH tetramer, unlike TH that only binds two DNAJC12s per tetramer (Table 1). In accordance with these results, we also analyzed samples containing PAH or TH with increasing amounts of DNAJC12 (4:1, 4:2, or 4:4) by native PAGE. In both cases, the complexes migrated slower through the gel than their unbound counterparts, but in the PAH samples combined with DNAJC12 at a 4:4 PAH:DNAJC12 ratio, unbound PAH still remained, differently from TH, for which full complex formation had already been achieved under the same conditions (Figure 2F). While TH and activated PAH share structural similarities, it is evident that DNAJC12 binds to both proteins at different stoichiometries.

**TABLE 1:** SEC‐MALS analysis of molecular masses and oligomeric states of TH and PAH, and their respective complexes with DNAJC12.

Proteins	Stoichiometry	Molecular mass (kDa)	Source
PAH	8(PAH)	383.1 ± 5.4	This work
	4(PAH)	194.1 ± 4.6	This work
PAH and DNAJC12	8(PAH):8(DNAJC12)	564.0 ± 4.6	This work
	4(PAH):4(DNAJC12)	286.2 ± 1.8	This work
MBP‐PAH(33–112)	2(MBP‐PAH(33–112))	101.2 ± 0.8	This work
MBP‐PAH(33–112) and DNAJC12	2(MBP‐PAH(33–112)):2(DNAJC12)	141.1 ± 1.1	This work
TH	4(TH)	217.8 ± 1.4	Tai et al., 2025
TH and DNAJC12	4(TH):2(DNAJC12)	266.1 ± 2.4	Tai et al., 2025
DNAJC12	1(DNAJC12)	22.7 ± 0.14	Tai et al., 2025

Although PAH is predominantly tetrameric, previous reports have indicated that PAH can form a minor population of octamers, potentially arising from inter‐tetramer interactions [30]. Indeed, we detected a small fraction of octameric PAH by SEC‐MALS (molecular mass 383.1 ± 5.4 kDa; Figure 2E), which interestingly also seems to bind DNAJC12, as observed by the appearance of species of 564.0 ± 4.6 kDa in the SEC‐MALS of the complex, corresponding to the binding of eight DNAJC12 monomers to each PAH octamer (Table 1, Figure 2E; stippled boxes). Evidence for this octameric species and its complex with DNAJC12 is also seen in native gels and immunoblots, where it appears as a slower‐migrating species than the predominant tetrameric PAH:DNAJC12 complex and is detected by both anti‐PAH and anti‐DNAJC12 antibodies (Figure 2C,F).

### 3.2 DNAJC12 Stabilizes PAH by Binding to the PAH‐RDs Through Its C‐Terminal Client Binding Domain (CTD)

**FIGURE 3:**
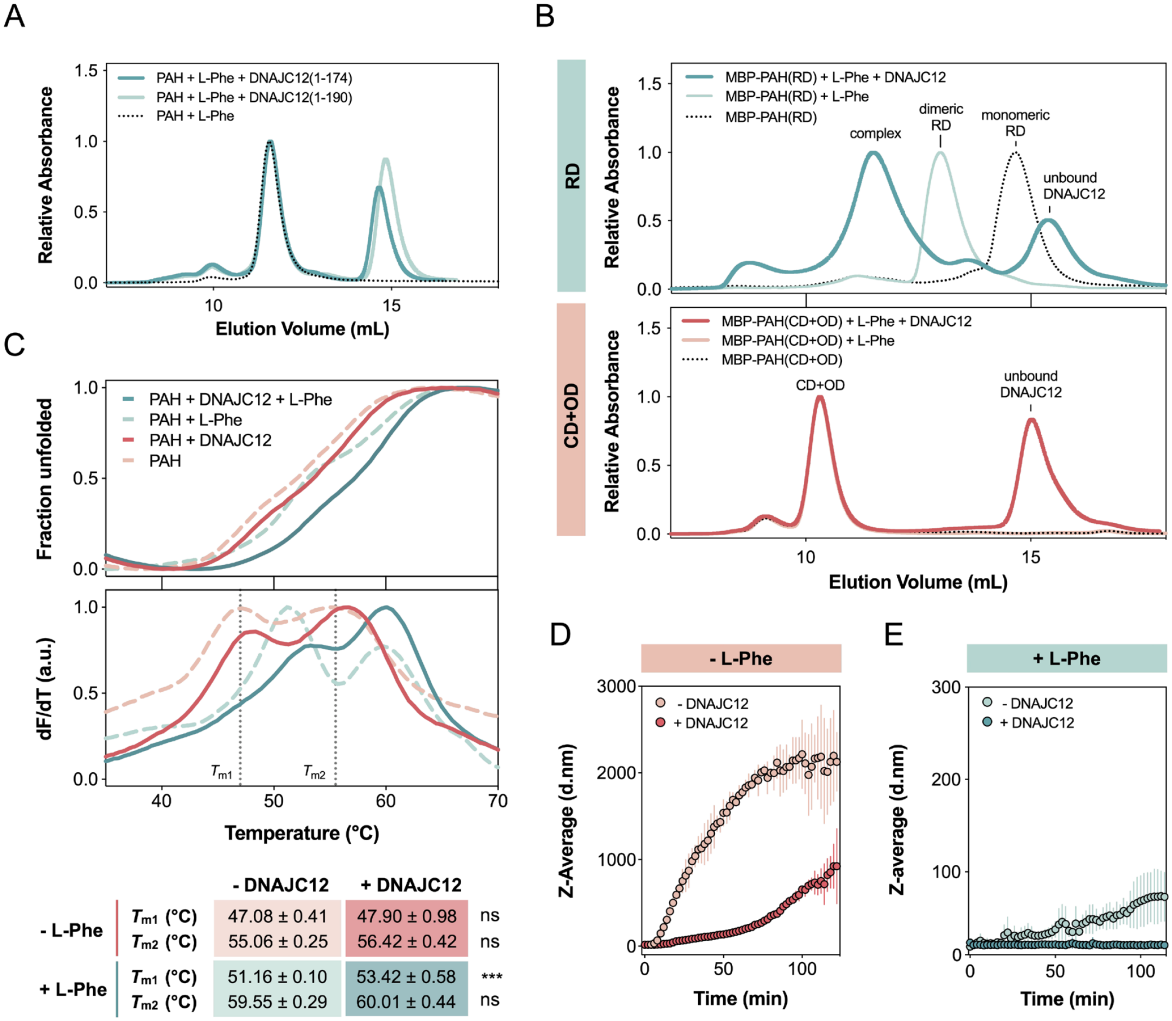
DNAJC12 binds and stabilizes the PAH‐RD. (A) SEC chromatograms of PAH alone and with C‐terminal truncated DNAJC12 variants, in the presence of 1 mM L‐Phe. DNAJC12(1–174) (dark blue; 40 μM) or DNAJC12(1–190) (light blue; 40 μM) do not affect the elution of PAH (stippled line; 20 μM subunit). (B) SEC chromatograms of MBP‐PAH(RD) or MBP‐PAH(CD + OD) alone and with 1 mM L‐Phe; effect of DNAJC12 binding. SEC analysis of MBP‐PAH(RD) (20 μM subunit; top) or MBP‐PAH(CD + OD) (20 μM subunit; bottom) in the presence (dark color line) or absence (light color line) of DNAJC12 (40 μM), without (stippled lines) or with 1 mM L‐Phe (solid lines). (C) DSF‐monitored unfolding. Representative thermograms for PAH (1.92 μM subunit) alone (stippled and light‐colored lines) or in presence of 3.84 μM DNAJC12 (solid and dark‐colored lines) with (blue) or without 1 mM L‐Phe (pink), showing the biphasic transition of PAH unfolding, corresponding to the denaturation of the RD (*T*_m1_) and the CD + OD (*T*_m2_). *T*_m_ values are presented as mean ± SD (*n* = 3 independent experiments) and analyzed by one‐way ANOVA and Tukey's post hoc HSD test to evaluate statistical differences in PAH *T*_m1_ or *T*_m2_ values in the presence or absence of DNAJC12 (***p = 0.0008). (D and E) DLS‐monitored aggregation of PAH in the presence and absence of DNAJC12, without (D) or with (E) 1 mM L‐Phe. Z‐average hydrodynamic diameter (d.nm; mean ± SD; *n* = 3 independent samples) of initially tetrameric PAH (10 μM subunit), monitored for 115 min at 37°C without (light symbols) and with DNAJC12 (10 μM; dark symbols) in the absence (red symbols) or presence of L‐Phe (blue symbols).

The DNAJC12 variant c.524G>A (p.W175Ter, also referred to as DNAJC12(1–174)) lacks the C‐terminal 23 amino acids [25] that includes the evolutionarily‐conserved heptapeptide sequence ^192^KFRNYEI^198^. This is the most common disease‐associated DNAJC12 variant identified to date [31] and has recently been shown to be unable to bind to TH, enabling the identification of this region (also known as the C‐terminal client‐binding domain (CTD); residues 176–198) as an essential determinant for TH:DNAJC12 complex formation [23]. To investigate whether DNAJC12 also binds to PAH using the same region, we purified the truncated DNAJC12 variants DNAJC12(1–174) and DNAJC12(1–190), and tested whether they could bind to PAH using the same SEC binding assay where the buffer was supplemented with 1 mM L‐Phe (Figure 3A). In contrast to the results obtained with full‐length DNAJC12 (Figure 2A; right), both truncated DNAJC12 variants do not alter the elution of PAH, indicating the necessity of the CTD for interaction with PAH (Figure 3A).

To probe whether DNAJC12 binds to the PAH‐RD, as observed for TH [23], we purified the PAH‐RD and CD + OD separately and analyzed binding by SEC. Due to the propensity of PAH‐RD to aggregate [7], we resorted to using the PAH(RD) or PAH(CD + OD) fused to a maltose binding protein (MBP) tag for the SEC binding experiments. The presence of MBP stabilizes the PAH(RDs) without inhibiting dimerization, as shown by the L‐Phe‐induced up‐shift of MBP‐PAH(RD) compared with the control without L‐Phe or DNAJC12. A further up‐shift in the elution of MBP‐PAH(RD) was observed in the presence of DNAJC12 and L‐Phe, indicating complex formation (Figure 3B; top panel). On the other hand, the presence of DNAJC12 or L‐Phe did not affect the elution profile of MBP‐PAH(CD + OD), confirming that DNAJC12 recognizes the PAH‐RD and not the CD + OD (Figure 3B; bottom panel). Subsequent SEC‐MALS analysis of MBP‐PAH(RD) (theoretical monomeric MW = 52.9 kDa) in the presence or absence of DNAJC12 (Table 1; Figure S1) showed that MBP‐PAH(RD) forms a dimer (101.2 ± 0.8 kDa) in 1 mM L‐Phe, and that addition of DNAJC12 further increases its molecular mass to ~141 ± 1.1 kDa. This ~40 kDa increase in molecular mass is consistent with two DNAJC12 molecules binding per PAH‐RD dimer, corresponding well with our earlier SEC‐MALS analyses with the full‐length PAH:DNAJC12 complex (Table 1; Figure 2E).

We then tested whether DNAJC12 exerted a stabilizing effect on PAH upon their interaction. Using differential scanning fluorimetry (DSF), we monitored the thermal unfolding of PAH in the presence or absence of DNAJC12 with 1 mM L‐Phe. Full‐length PAH displays a biphasic transition (Figure 3C) corresponding to the sequential denaturation of the RD (*T*_m1_ = 47.08°C ± 0.41°C) and the CD + OD (*T*_m2_ = 55.06°C ± 0.25°C) [32] and thus, it was possible to monitor the effect of DNAJC12 on these domains by comparing the obtained melting temperatures (*T*_m1_ and *T*_m2_, corresponding to the denaturation of the RD and the CD + OD, respectively) in the presence and absence of DNAJC12, with or without L‐Phe. Corresponding well with previous reports that demonstrate the thermal stabilization of the PAH‐RD induced by the presence of L‐Phe, we observed a 4°C increase in *T*_m1_, from 47.08°C ± 0.41°C without L‐Phe, to 51.16°C ± 0.10°C with 1 mM L‐Phe, which is related to its dimerization in response to substrate‐induced activation [7]. A similar increase in *T*_m2_ was observed, indicating the stabilization of the CD + OD upon the addition of 1 mM L‐Phe, consistent with the binding of L‐Phe to the active site. In agreement with our SEC results showing that DNAJC12 binds to the MBP‐PAH(RD) and not MBP‐PAH(CD + OD) (Figure 3B), we found that the presence of DNAJC12 did not significantly increase the *T*_m2_ of PAH regardless of the presence or absence of L‐Phe (Figure 3C). Interestingly, however, a 2°C increase in PAH *T*_m1_ was observed in the presence of DNAJC12 with 1 mM L‐Phe (*T*_m1_ = 53.42°C ± 0.58°C) as compared to when the enzyme is alone (*T*_m1_ = 51.16°C ± 0.10°C), while no significant changes were observed when there was no L‐Phe (*T*_m1_ = 47.08°C ± 0.41°C for PAH alone; *T*_m1_ = 47.90°C ± 0.98°C for PAH with DNAJC12). Thus, in the presence of L‐Phe DNAJC12 serves as a reporter of the PAH‐RDs, and its binding results in the stabilization of these domains. Additional DSF assays monitoring the thermal denaturation of full‐length PAH in the presence of increasing concentrations of the C‐terminal DNAJC12 peptide, DNAJC12(176–198), likewise showed a dose‐dependent stabilization of *T*_m1_, consistent with peptide binding and its stabilizing effect on the PAH‐RDs (Figure S2).

To assess whether the thermal stabilization of PAH also resulted in a delay in its aggregation over time in vitro, we monitored changes in the particle size of PAH with and without DNAJC12, both in the absence and presence of 1 mM L‐Phe, for 115 min. Consistent with the significant thermal stabilization imparted by L‐Phe to PAH (Figure 3C), we observed that the presence of the substrate significantly delays PAH aggregation in vitro, reducing the particle size at the end of the recording from 2022 ± 604 nm without L‐Phe to 73 ± 26 nm with L‐Phe (Figure 3D,E). Additionally, DNAJC12 shows protection towards PAH aggregation even without L‐Phe (716 ± 234 nm particle size; Figure 3D), but is even more effective when L‐Phe is present, leading to a remarkable reduction of the aggregation (21 ± 2 nm particle size; Figure 3E), which seems related to the increased affinity and saturation of DNAJC12 binding to the substrate activated PAH (Figure 2A,D). These findings collectively highlight the stabilizing effect of DNAJC12 on PAH, particularly when combined with L‐Phe.

### 3.3 DNAJC12 Decreases the L‐Phe Concentration Required for Enzyme Activation

**FIGURE 4:**
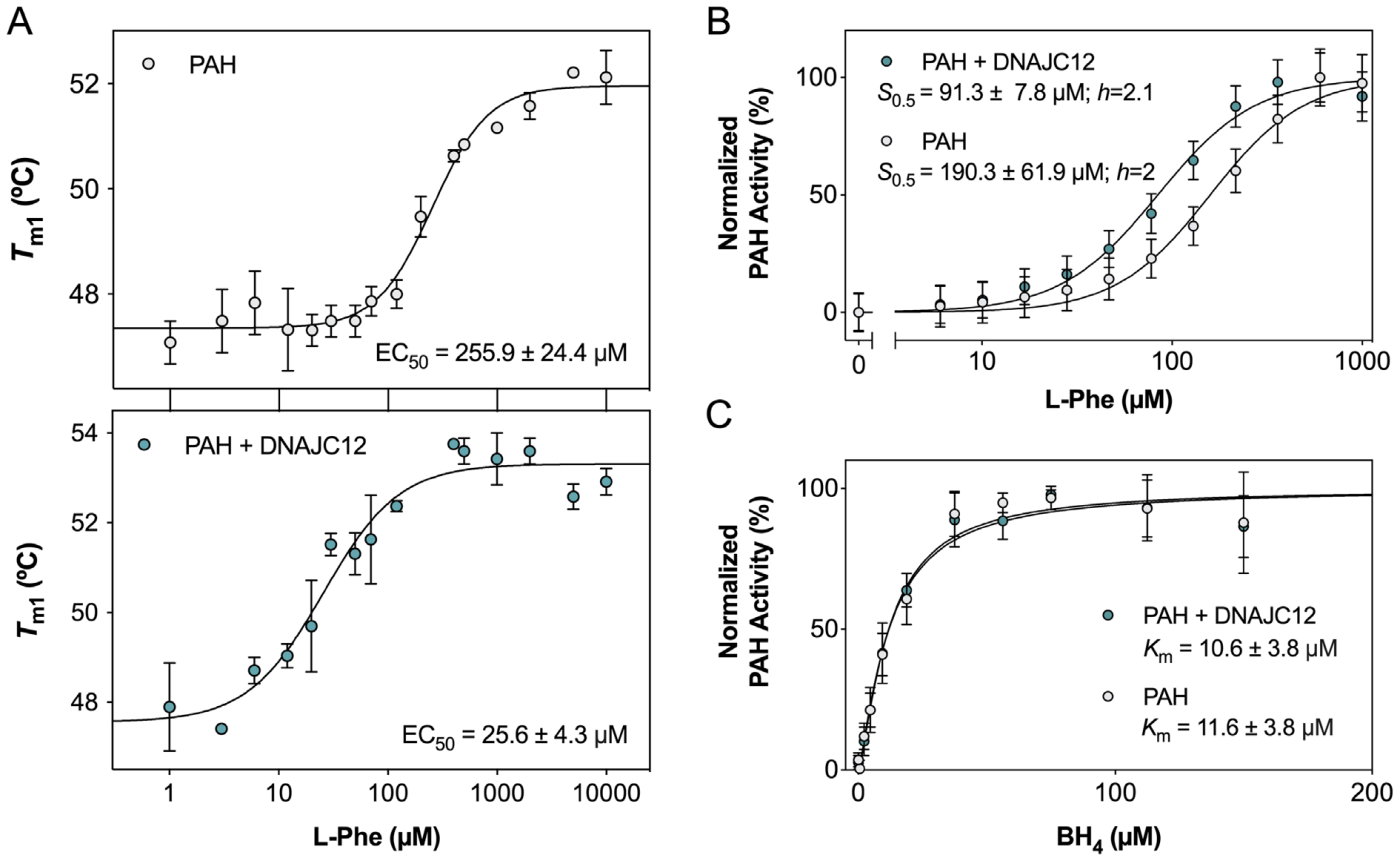
DNAJC12 lowers the L‐Phe concentration necessary for substrate‐induced activation. (A) Concentration‐dependent stabilization of PAH by L‐Phe without (upper panel) and with DNAJC12 (lower panel). The thermal unfolding of PAH‐RD at varying L‐Phe concentrations (0–10 000 μM) with (bottom) and without (top) excess DNAJC12 (4:8 PAH:DNAJC12 subunit ratio) was monitored by following the first melting temperature transition (*T*_m1_) of full‐length PAH. Fitting the data to a four‐parameter logistic nonlinear regression provides EC_50_ values (mean ± SD; *n* = 3 independent samples). (B and C) Enzymatic activity of PAH at variable L‐Phe (0–1000 μM; B) and BH_4_ concentrations (0–150 μM; C). (B) The L‐Phe concentration dependent activity of PAH presents positive cooperativity, with the indicated half‐maximal activity (*S*_0.5_) and Hill coefficient (*h*). The presence of DNAJC12 maintains the positive cooperativity (*h* = 2) but increases the affinity of PAH for L‐Phe, as indicated by the 54% reduction in *S*_0.5_. (C) The BH_4_ concentration dependence of PAH activity follows Michaelis–Menten kinetics, and DNAJC12 does not alter the *K*_m_ value for the cofactor. Activity data are presented as normalized results, *n* = 3 independent experiments, each with technical triplicates, fitted to a four‐parameter dose–response curve. *S*_0.5_ and *K*_m_ values are presented as mean ± SD.

To investigate whether DNAJC12 binding affects the L‐Phe dependent regulation of PAH and/or the enzyme kinetic parameters, we first monitored the thermal unfolding of full‐length PAH in the presence or absence of DNAJC12 using DSF at varying L‐Phe concentrations. Since DNAJC12 binds to the PAH‐RDs, which are stabilized upon L‐Phe binding (Figure 3B,C), we monitored any changes in *T*_m1_ values, which correspond to the unfolding of the PAH‐RDs, at varying L‐Phe concentrations. These *T*_m1_ values were plotted and fitted to a four‐parameter curve, providing the substrate concentration at which half‐maximal stabilization of this domain is reached (EC_50_). We found that the EC_50_ for the PAH‐RD is approximately 10 times higher (255.9 ± 24.4 μM) when PAH is alone than in the presence of DNAJC12 (25.6 ± 4.3 μM) (Figure 4A, top and bottom panels), indicating that the binding of DNAJC12 to PAH lowers the concentration of L‐Phe required to stabilize the dimeric PAH‐RDs that characterize the L‐Phe activated PAH state (Figure 1).

To determine whether the observed combined stabilization of the dimeric RDs by L‐Phe and DNAJC12 results in a lower L‐Phe concentration necessary to elicit the cooperative conformational changes during PAH activation, we measured PAH activity in the presence and absence of DNAJC12 at varying L‐Phe concentrations. Consistent with previous studies [8, 9], we found that full‐length PAH wild‐type (PAH‐WT) alone presents positive cooperativity for L‐Phe providing half‐maximal activity (*S*_0.5_) at 190.3 ± 61.9 μM (Hill coefficient; *h* = 2). Interestingly, in the presence of DNAJC12, a ~54% decrease in the PAH *S*_0.5_ to 91.3 ± 7.8 μM was obtained (Figure 4B), without affecting the positive cooperativity (*h* = 2.1), indicating that DNAJC12 increases PAH activity at lower substrate concentrations. In addition, PAH activity measurements at a range of BH_4_ concentrations show the hyperbolic dependence of the activity vs. BH_4_ concentration and that DNAJC12 does not affect the *K*_m_‐value for the cofactor (Figure 4C). Together, these findings indicate that DNAJC12 lowers the L‐Phe concentration needed to stabilize dimerized PAH‐RDs and, consequently, the activated conformation of PAH, thereby enabling maximal enzymatic activity without affecting the affinity of PAH for BH_4_.

### 3.4 DNAJC12 Also Binds and Stabilizes the PKU‐Associated Variant PAH‐p.R261Q and Delays Its Aggregation In Vitro

**FIGURE 5:**
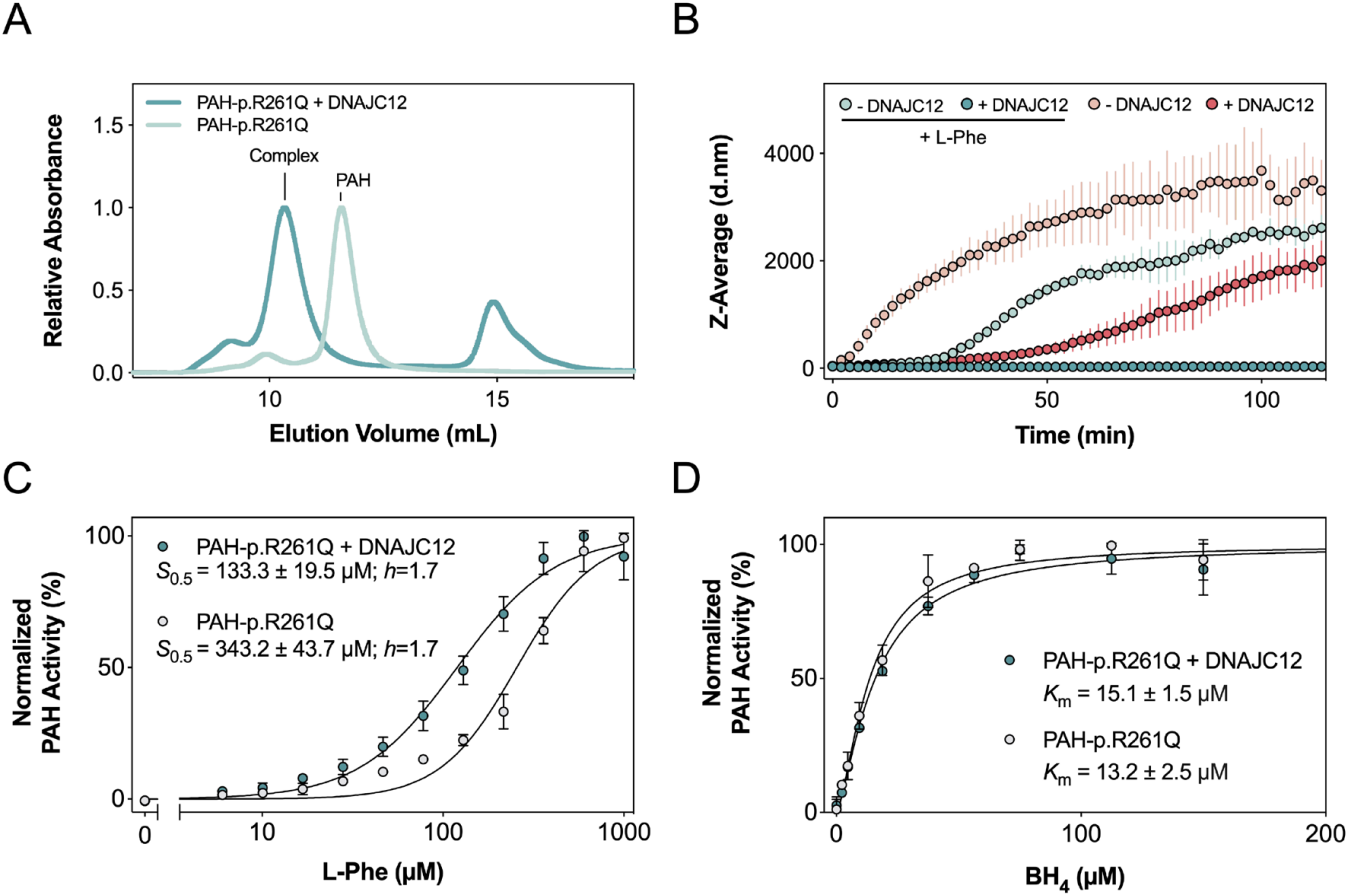
DNAJC12 binds and stabilizes the PKU‐associated variant PAH‐p.R261Q, and decreases the L‐Phe concentration necessary for its enzymatic activation. (A) SEC chromatograms of PAH‐p.R261Q alone and in the presence of DNAJC12. Excess DNAJC12 (25 μM) shifts the elution of tetrameric PAH‐p.R261Q (20 μM subunit) in the presence of 1 mM L‐Phe, indicating efficient complex formation. (B) DLS‐monitored aggregation of PAH‐p.R261Q with and without DNAJC12 or L‐Phe. Z‐average hydrodynamic diameter (d.nm; mean ± SD; *n* = 3 independent samples) of initially tetrameric PAH‐p.R261Q (10 μM subunit), for 115 min at 37°C without (light symbols) and with DNAJC12 (10 μM; dark symbols), without (red symbols) or with 1 mM L‐Phe (blue symbols). (C and D) Enzymatic activity of PAH‐p.R261Q at variable L‐Phe (0–1000 μM; C) and BH_4_ concentrations (0–150 μM; D). (C) The L‐Phe concentration dependent activity of PAH presents positive cooperativity, with the indicated half‐maximal activity (*S*_0.5_) and Hill coefficient (*h*). The presence of DNAJC12 does not affect the *h*‐value (*h* = 1.7) but increases the affinity of PAH‐p.R261Q for L‐Phe, as indicated by the 65% reduction in *S*_0.5_. (D) The BH_4_ concentration dependence of PAH‐p.R261Q activity follows Michaelis–Menten kinetics, and DNAJC12 does not alter the *K*_m_ value for the cofactor. Activity data are presented as normalized results, *n* = 3 independent experiments, each with technical triplicates, fitted to a four‐parameter dose–response curve. *S*_0.5_ and *K*_m_ values are presented as mean ± SD.

We tested whether the misfolded and unstable PKU‐associated variant PAH‐p.R261Q can also be recognized by DNAJC12 as a client protein. Using SEC, we found that DNAJC12 can form a complex with purified PAH‐p.R261Q in the presence of L‐Phe (Figure 5A). This variant has been shown to form aggregates both in vitro and in the liver of a mouse model [17], and thus, we tested if the stabilization imparted by DNAJC12 also translates to a delay in its aggregation. By monitoring the changes in PAH‐p.R261Q particle size over 115 min, we observed that initially tetrameric PAH‐p.R261Q forms aggregates over time (3309 ± 561 nm at 115 min), and to a lesser extent in the presence of L‐Phe (2618 ± 233 nm at 115 min), indicating that the variant responds similarly to PAH‐WT to substrate‐dependent stabilization, although this protective effect appears to be smaller in PAH‐p.R261Q (Figure 5B) than in PAH‐WT (Figure 3D,E). Consistent with our results with PAH‐WT, we found that DNAJC12 delays the aggregation of PAH‐p.R261Q, even in the absence of L‐Phe (2007 ± 486 nm at 115 min), but with even better efficacy in the presence of L‐Phe (114 ± 1 nm at 115 min) (Figure 5B).

We also measured the conversion of L‐Phe to L‐Tyr by PAH‐p.R261Q, in the presence and absence of DNAJC12, at varying concentrations of L‐Phe or BH_4_. Consistent with previous studies that report that this variant also presents positive cooperativity, with a somehow increased *S*_0.5_ compared with PAH‐WT [14], we measured a *S*_0.5_ = 343.2 ± 43.7 μM L‐Phe (Figure 5C), around double the *S*_0.5_ of PAH‐WT (Figure 4B), and a *h* = 1.7. Remarkably, in the presence of DNAJC12, the *S*_0.5_ value of PAH‐p.R261Q decreased by ~65%, to 133.3 ± 19.5 μM L‐Phe (Figure 5C). As with PAH‐WT, DNAJC12 also does not affect the *K*_m_‐value of BH_4_ for PAH‐p.R261Q (Figure 5D).

### 3.5 PAH and DNAJC12 Synergistically Activate Hsc70 ATPase Activity With L‐Phe

**FIGURE 6:**
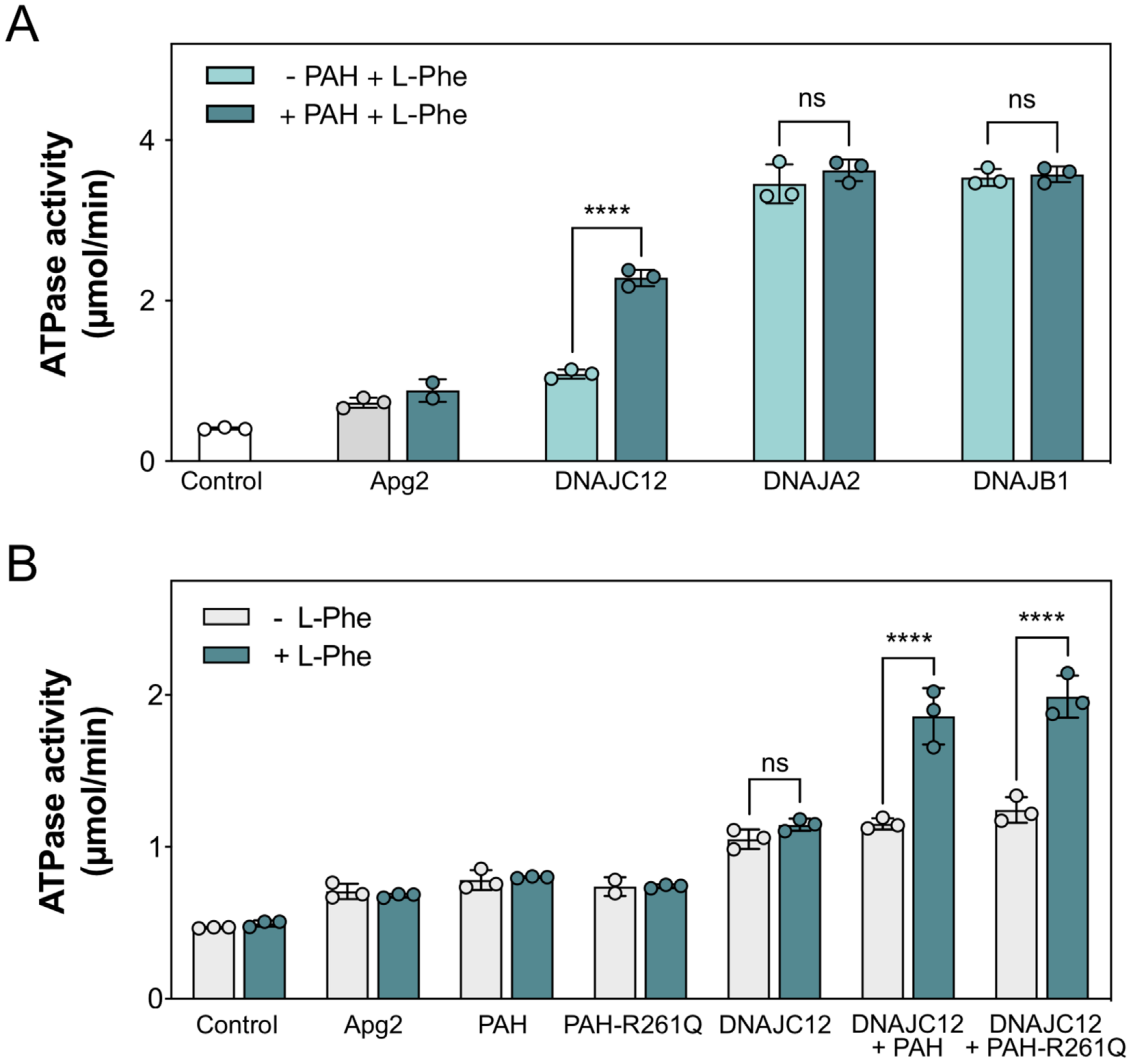
Hsc70 ATPase activity stimulation by DNAJC12 compared to model JDPs, in the presence and absence of L‐Phe and/or PAH. (A) Stimulation of Hsc70 ATPase activity by JDPs with and without PAH, with 1 mM L‐Phe. Hsc70 ATPase activity was measured in the presence of DNAJC12 and the canonical JDPs DNAJA2 and DNAJB1, with (dark green) and without (light green) PAH. Data shown represent the mean ± SD for *n* = 3 independent experiments. The ATPase activity recorded in the presence of PAH was compared to that recorded in the absence of the enzyme, by one‐way ANOVA and Tukey's post hoc HSD test (****p < 0.0001). (B) Stimulation of Hsc70 ATPase activity by DNAJC12 in the presence or absence of PAH, with and without 1 mM L‐Phe. The ability of DNAJC12 to stimulate Hsc70 ATPase activity was measured with and without PAH (WT or p.R261Q), with (dark green) or without (gray) L‐Phe. Data shown here represent the mean ± SD for *n* = 3 independent experiments. The ATPase activity recorded with L‐Phe was compared to their respective controls measured without L‐Phe, by one‐way ANOVA and Tukey's post hoc HSD test (****p < 0.0001).

We have previously shown that as expected from a JDP, DNAJC12 activates Hsc70 ATPase activity, though at much lower levels compared to canonical JDPs DNAJA2 and DNAJB1 [23], as also shown in Figure 6A. Moreover, as seen in Figure 6B, L‐Phe alone did not stimulate its activity. We investigated whether PAH and DNAJC12 could synergistically activate Hsc70 ATPase activity, with or without L‐Phe. We found that PAH or PAH‐p.R261Q alone do not stimulate Hsc70 ATPase activity, regardless of the presence of L‐Phe (Figure 6B), and the addition of PAH or PAH‐p.R261Q only slightly improves DNAJC12 activity. However, the combination of either of these PAHs with L‐Phe significantly enhances the stimulatory effect of DNAJC12 on Hsc70 ATPase activity (Figure 6B), supporting the importance of L‐Phe for PAH:DNAJC12 complex formation. This effect appears specific to DNAJC12, as the same was not observed with canonical JDPs DNAJA2 and DNAJB1, whose stimulatory effect on Hsc70 ATPase activity remains unaffected by PAH addition, even with L‐Phe (Figure 6A).

## 4 Discussion

An extensive network of molecular chaperones cooperates to maintain protein homeostasis. Thus, mutations in chaperones dysregulate these networks and may consequently affect the proteostasis of their client proteins, often leading to disease. Variants of the JDP *DNAJC12* cause HPA, dystonia, intellectual disabilities and neurotransmitter deficiencies, revealing the importance of this cochaperone in the regulation of the AAAHs [19, 20, 21]. The role of DNAJC12 in the maintenance of AAAH homeostasis has since been corroborated [22, 24, 25], and characterization of the interaction between TH and DNAJC12 has recently improved the understanding of the determinants for DNAJC12‐client binding [23]. However, little was known about the underlying molecular mechanisms or the regulatory and functional consequences of the PAH:DNAJC12 interaction. In this work, we identified the binding determinants mediating this association and demonstrated how this protein–protein interaction affects PAH stability and enzymatic activity.

Allosteric cooperativity is a key regulatory mechanism commonly found in metabolic and signaling pathways, and is usually mediated by ligand‐induced conformational changes [33]. PAH is known to exist in two conformational states depending on the concentration of its substrate, L‐Phe [1, 11, 12]. At low substrate concentrations, the low activity conformation predominates, with monomeric RDs where the autoregulatory tail (residues 1–29) partly occludes the PAH active site [5]. Increasing L‐Phe concentrations shift the conformational equilibrium toward the activated state, which becomes dominant at L‐Phe levels ≥ 1 mM. Activated PAH is characterized by the dimerization of PAH‐RDs from opposite subunits in the tetramer [11, 12]. The structure of the dimeric RDs with L‐Phe bound at the interface has been solved using X‐ray diffraction crystallography [7], but the high‐resolution structure of the full‐length substrate‐bound PAH remains undetermined. In addition, the mechanism of substrate‐induced activation remains unclear, but it has been proposed that L‐Phe binding to the allosteric site in the RDs and consequent dimerization of these domains would release the inhibitory interactions of the autoregulatory tail (residues 1–29), which partly occludes the PAH active site in the autoinhibited state [11, 12, 34]. Although the large activating conformational change increases PAH activity and stabilizes the tetramer [12], the inherent dynamics necessary for maintaining high activity in this state may be challenging. Hence, increased interaction of DNAJC12 with activated PAH might have an implication on the overall stability of the enzyme in the activated state.

PAH:DNAJC12 complex formation was remarkably improved in the presence of L‐Phe (Figure 2A,D), indicating that the substrate‐induced active conformation of PAH is likely a better physiological target for DNAJC12 than unactivated PAH. To our knowledge, TH has not been reported to be allosterically activated by its substrate L‐Tyr or by other amino acids, in contrast to PAH. Consequently, TH does not undergo ligand‐induced conformational rearrangements associated with the regulatory domain, and instead displays a distinct dimeric RD organization, comparable to that of activated PAH (Figure 1) [6, 35]. Interestingly, DNAJC12 binds unliganded TH effectively [23], with an affinity similar to that measured for activated PAH (K_D_ = 139 ± 19 nM). The full‐length structures of the other two AAAHs, TPH1 and TPH2, are still undetermined and it is still uncertain whether L‐Trp or other aromatic amino acids could activate these enzymes [4], thus it is unknown whether DNAJC12 can readily bind to the unliganded forms of these enzymes as it does with TH.

**FIGURE 7:**
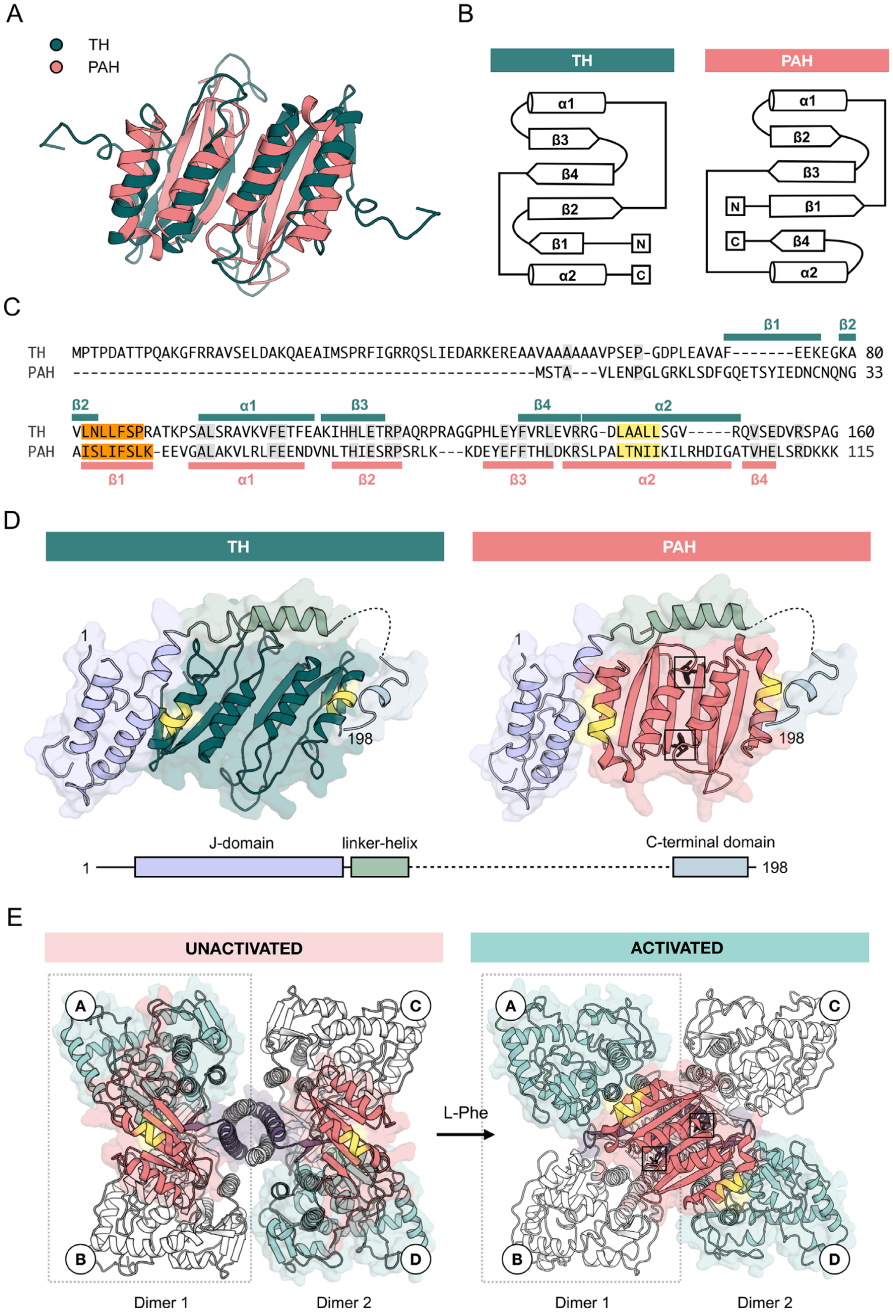
The modeled structure of activated PAH with the proposed PAH:DNAJC12 interaction region. (A) Structural comparison of the dimeric TH and PAH RDs. Overlay of the human TH‐RD solved by cryo‐EM (PDB 6ZVP [6]; green) and of human PAH‐RD with L‐Phe solved by X‐ray crystallography (PDB 5FII [7]; red) using PyMol. (B) Topology diagrams of the TH (green) and PAH RDs (red). The secondary structure elements of both the TH and PAH RDs are shown in the figure. (C) Sequence alignment of the TH and PAH RDs. The sequence alignment, by ClustalOmega, and the secondary structures were mapped on the sequence based on the TH (PDB 6ZVP [6]; green) and PAH‐RD (PDB 5FII [7]; red) structures. The region determined by Tai et al. [23] in the TH‐RD (^141^LAALL^145^) that interacts with DNAJC12, and the equivalent sequence in PAH (^91^LTNII^95^) are highlighted in yellow, while the TANGO [36] ‐predicted residues in both TH (^82^LNLLFSP^88^) [23] and PAH (^35^ISLIFSLK^42^), with high aggregation propensity, are highlighted in orange. (D) Model of PAH‐RD:DNAJC12 binding. The dimeric PAH‐RD crystal structure (PDB 5FII [7]; right) was aligned to the TH RD in the TH:DNAJC12 model that was developed based on cryo‐EM, crosslinking‐mass spectrometry and in silico analyses [23] (left). The J‐domain is highlighted in purple, the linker‐helix in green and the client‐binding CTD in blue. The proposed interaction region is colored in yellow in both models and the two L‐Phe molecules bound in the dimeric PAH‐RD are highlighted with a black box. A tentative model showing the dimeric PAH‐RD bound to two DNAJC12 molecules is shown in Figure S3. (E) Model showing conformational changes in PAH induced by substrate binding. Crystal structure of full‐length unactivated human PAH (PDB 6HYC [5]; left) and a composite model of activated full‐length PAH (right) made from the crystal structures of human CD + OD (PDB 2PAH) and human PAH RD (PDB 5FII [7]), with L‐Phe bound on the dimeric interface (black box). The RD is colored in red, CD in blue, and OD in purple. The site proposed to be involved in DNAJC12 interaction is in yellow.

By combining cryo‐EM, crosslinking mass spectrometry, and site directed mutagenesis, we previously identified key residues in TH that are likely involved in complex formation with DNAJC12 [23]. TH alanine variants at L141 and L145 exhibit improved affinity for DNAJC12 due to better packing, substantiating the involvement of these residues in hydrophobic interactions with the DNAJC12 CTD [23]. While the TH and PAH RDs share high structural homology (Figure 7A), they differ in folding (Figure 7B) and sequence (Figure 7C). By aligning sequences and mapping the different secondary structures in the RDs of both proteins based on solved structures of human dimeric PAH‐RD (PDB 5FII) [7] and TH‐RD (PDB 6ZVP) [6], we found that the sequence that is involved in TH binding to DNAJC12 (^141^LAALL^145^) [23] aligns with the rather similar sequence ^91^LTNII^95^ in PAH (Figure 7C,D). The hydrophobic Leu residues involved in the interaction of TH with the DNAJC12 CTD [23] appear preserved as Leu/Ile. This region is quite exposed in unactivated PAH (Figure 7E; left), but it may be possible that the orientation of the RDs and steric hindrances imposed by the central CD + OD in this state prevents the efficient binding of DNAJC12, and explains the low‐affinity interaction between DNAJC12 and unactivated PAH, as seen by the SEC, binding, DSF and aggregation assays without L‐Phe. In the activated state, these residues are exposed in the same orientation observed in the analogous region in TH (Figure 7D,E), and could explain why the affinity of DNAJC12 increases for activated PAH, to a similar level as previously reported for the TH:DNAJC12 interaction (K_D_ = 148 nM) [23]. In addition, the 4:4 DNAJC12 monomer:PAH subunit stoichiometry indicates that two DNAJC12 monomers could bind to each RD dimer, potentially oriented in opposite directions to stabilize the interaction, as shown in the tentative structural model shown in Figure S3. This conformation may facilitate the retention of L‐Phe within the RD, and potentially preserve some L‐Phe to avoid depleting the amino acid during enzyme activation. However, the structural details and functional impact of PAH:DNAJC12 complex formation remain to be explored experimentally.

In addition to their role as cochaperones of Hsp70, JDPs may directly protect regions in their client proteins that have a high propensity for aggregation by a holdase activity [37, 38]. A similar protective effect has been reported for DNAJC12 in its interaction with TH, preventing TH aggregation in vitro, which seems related by the covering of the aggregation prone residues ^82^LNLLFSP^88^ in the β1‐strand in the TH‐RD by DNAJC12 binding [23]. Similarly, the residues ^35^ISLIFSLK^42^ lying in the β1‐strand of the PAH‐RD are predicted by TANGO [36] to have high propensity to engage in intermolecular cross‐β interactions, and may also be covered by DNAJC12 binding (Figure 7D). The binding of DNAJC12 to the dimeric RDs could further contribute to prevent aggregation of PAH‐WT and PAH‐p.R261Q by avoiding the dissociation of the dimers into monomers, a process that typically precedes aggregation [39, 40]. L‐Phe itself stabilizes PAH [7] and facilitates RD dimer formation from different PAH dimers [11, 41], resulting in an additional tetramerization interface apart from the oligomerization domain (Figure 7E). The strategic positioning of DNAJC12 could thus be especially valuable to stabilize aggregation‐prone and unstable PAH variants such as PAH‐p.R261Q. This substitution from Arg to Gln at position 261 is predicted to disrupt the interdimer interactions in PAH‐p.R261Q and, consequently, the region surrounding the mutation may become prone to nonspecific intersubunit interactions [17]. Although DNAJC12 seems efficient in delaying the formation of PAH‐p.R261Q aggregates in vitro, toxic amyloid‐like PAH‐p.R261Q aggregates have been identified in a mouse model despite the presence of DNAJC12‐WT [17]. While our in vitro studies show that the binding stoichiometry between PAH and DNAJC12 is 1:1 per subunit, the expression of PAH mRNA has been reported to be approximately 20‐fold higher than DNAJC12 in human liver samples [42] (https://www.proteinatlas.org/). The disparity between expression levels could explain why DNAJC12 is unable to effectively prevent PAH‐p.R261Q aggregation in the mouse model. All in all, our in vitro experiments contribute to the understanding of DNAJC12 function and demonstrate the potential of increasing DNAJC12 expression or enhancing PAH:DNAJC12 interactions as a therapeutic strategy to stabilize and delay the aggregation of unstable PAH variants.

Previous in vitro characterizations have shown that preincubation of PAH with L‐Phe increases PAH activity, as the enzyme transitions into its activated catalytic state [8, 10, 43]. The L‐Phe preactivated enzyme exhibits half‐maximal activity at approximately 190 μM (Figure 4B), a concentration not typically reached in the blood of healthy individuals and already considered within the range of mild HPA [3, 44]. In this study, we show that DNAJC12 enhances PAH activity at low substrate concentrations by lowering the *S*_0.5_ for L‐Phe, without altering the positive cooperativity, thereby shifting PAH activation to a range more relevant for healthy individuals and revealing an additional layer of PAH regulation. The observed stabilization of PAH by DNAJC12 in the activated conformation may explain why the cochaperone increases PAH activity at lower L‐Phe concentrations and why patients with DNAJC12 deficiency often present with HPA as a consequence of both reduced activity and decreased stability [25]. Apart from these effects of DNAJC12 binding on PAH stability and activity, PAH:DNAJC12 complex formation seems to be essential for stimulation of Hsc70 ATPase activity by DNAJC12. Unlike better characterized JDPs such as DNAJA2 and DNAJB1, which are able to stimulate Hsc70 ATPase activity by themselves, without a client protein, DNAJC12 displays low stimulatory effect on its own, and synergistically stimulates Hsc70 ATPase activity when in complex with either TH [23] or with PAH in an L‐Phe dependent manner. These results suggest that the synergistic stimulation of Hsc70 ATPase activity by DNAJC12 may be preserved across its interactions with all four AAAH clients, notably in a dimerized RD conformation.

After several catalytic cycles, enzymes have a tendency to misfold or catalyze reactions inefficiently, often due to collateral damage from the reactions they facilitate [45], and a catalysis‐related loss of function due to generation of oxygen reactive species has indeed been shown for PAH [46, 47]. The binding of DNAJC12 to activated PAH could thus enable Hsc70‐mediated refolding or degradation to facilitate protein turnover. Overall, our findings suggest that DNAJC12 plays a crucial role in stabilizing PAH and enhancing its activity at lower L‐Phe levels, and also in maintaining PAH proteostasis, presenting the PAH:DNAJC12 interaction as a potential therapeutic target for HPA and PKU. Nevertheless, a long‐term stabilization of PAH in the active state by DNAJC12 should be properly regulated, as it could result in the undesirable depletion of L‐Phe, an essential amino acid required for protein synthesis that can contribute to amino acid–dependent regulation of mRNA translation [48, 49].

## Author Contributions

Mary Dayne S. Tai, Marte I. Flydal, and Christer F. Didriksen purified PAH and DNAJC12, including all truncated forms and variants used in this work, and prepared the PAH:DNAJC12 complexes. Mary Dayne S. Tai, Gloria Gamiz‐Arco, Marte I. Flydal and Christer F. Didriksen carried out the biophysical analyses, while Fernando Moro and Trond‐André Kråkenes performed biochemical experiments. Mary Dayne S. Tai, Marte I. Flydal, Juha P. Kallio, Fernando Moro and Aurora Martinez designed the experiments. All authors analyzed the data. Aurora Martinez managed the project and wrote the paper with main contributions from Mary Dayne S. Tai and corrections from all authors.

## Funding

This work was supported by Stiftelsen Kristian Gerhard Jebsen (KGJF), SKJ‐MED‐02. Norges Forskningsråd (Forskningsrådet), 288164 and 245922. Fundació la Marató de TV3 (Fundació la Marató), 202012‐31. L. Meltzers Høyskolefond (Meltzerfondet), 103578. Fondo Europeo de Desarrollo Regional, PID2023‐152081NB‐I00. Basque Government, IT1745‐22.

## Conflicts of Interest

The authors declare no conflicts of interest.

## Supporting information


**Figure S1:** fsb272151‐sup‐0001‐Supinfo.pdf.

## Data Availability

All data generated or analyzed during this study are included in this article and/or its Supporting Information files. Further enquiries can be directed to the corresponding author.
