# Supplementary material for: DNAJC12 Stabilizes Phenylalanine Hydroxylase and Facilitates Its Substrate‐Dependent Activation
Source: FASEB J. 2026 Jul 24;40(14):e72151. doi: 10.1096/fj.202504522RR (PMC13399020; doi:10.1096/fj.202504522RR)
Supplement: Supplementary file 1 — Figure S1: fsb272151‐sup‐0001‐Supinfo.pdf. [file FSB2-40-e72151-s001.pdf]

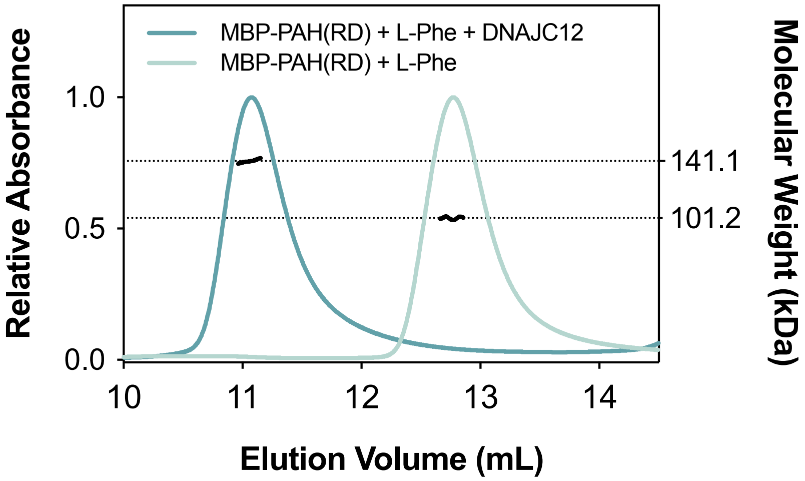

Supplementary Figure 1. SEC-MALS analysis of MBP-PAH(RD) in the presence (dark blue) or absence (light blue) of DNAJC12. SEC-MALS was performed on a Superdex 200 10/300 column pre-equilibrated with 20 mM Na-Hepes pH 7.0, 200 mM NaCl and 1 mM L-Phe. The MBP-PAH(RD) species was observed at 101.2 ± 0.8 kDa, consistent with dimer formation. The complex exhibited an additional ~40 kDa increase in molecular weight, suggesting the binding of two DNAJC12 molecules to the MBP-PAH(RD) dimer.

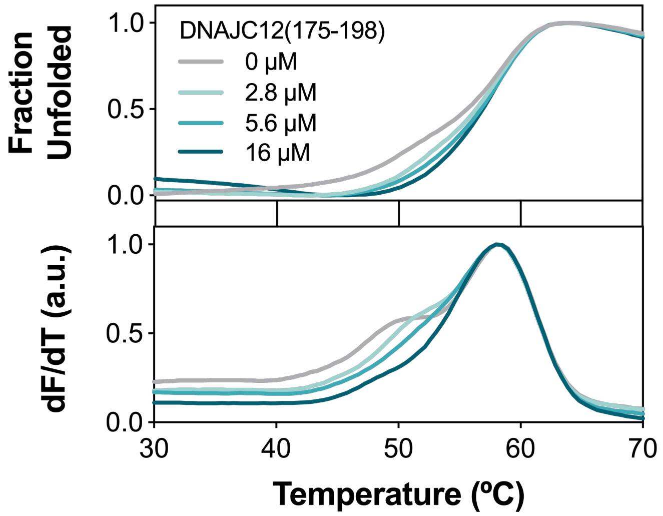

Supplementary Figure 2. DSF monitored analysis of PAH in the presence of varying concentrations (0-16 mM) of the DNAJC12(176-198) peptide. Upper panel, DSF thermograms (fluorescence vs. temperature) and lower panel, first derivative of the thermograms, used to estimate T_m. Alone, PAH presents a biphasic transition of denaturation, representing the sequential denaturation of the RD (T_{m1}=~51°C) and the CD+OD (T_{m2}=~58°C). The addition of increasing concentrations of the DNAJC12 peptide results in the dose-dependent stabilization of PAH, particularly through a thermal shift of T_{m1} but not T_{m2}, indicating that the peptide binds and stabilizes the RD, but not the CD+OD.

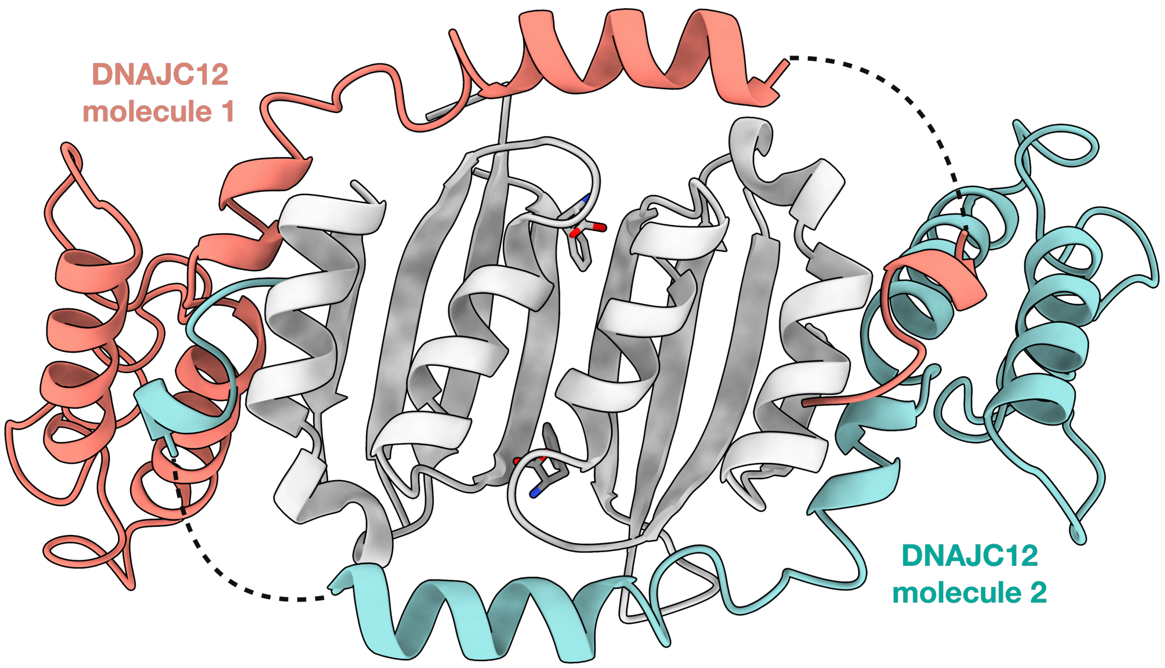

Supplementary Figure 3. Tentative model of two DNAJC12 molecules bound to the dimeric PAH regulatory domains (PAH-RDs). DNAJC12 monomers (AlphaFold model AF-Q9UKB3-F1; orange and blue) are docked onto the PAH-RD dimer (light gray) with L-Phe bound at the dimer interface. One DNAJC12 molecule was positioned by replacing the dimeric TH regulatory domain (TH-RD) in the TH:DNAJC12 complex[1] with PAH-RD (PDB 5FII)[2]. A second DNAJC12 molecule was placed on the opposite side by applying an approximate twofold (C2) symmetry about an axis perpendicular to the dimer interface. Minor manual adjustments were made to relieve steric clashes.

References

1. Tai, M.D.S. et al. Structural recognition and stabilization of tyrosine hydroxylase by the J-domain protein DNAJC12. *Nat Commun* **16**, 2755 (2025).
2. Patel, D., Kopec, J., Fitzpatrick, F., McCorvie, T.J. & Yue, W.W. Structural basis for ligand-dependent dimerization of phenylalanine hydroxylase regulatory domain. *Sci Rep* **6**, 23748 (2016).
